# Supplementary material for: Evaluation of intron-1 of odorant-binding protein-1 of Anopheles stephensi as a marker for the identification of biological forms or putative sibling species
Source: PLoS One. 2022 Jul 21;17(7):e0270760. doi: 10.1371/journal.pone.0270760 (PMC9302840; doi:10.1371/journal.pone.0270760)
Supplement: S2 Table — (DOCX) [file pone.0270760.s002.docx]

**S2** **Table.** Pairwise *F*_ST_ values between the three biological forms based on *AsteObp1* intron-1 sequences

|  | Type form | Intermediate | *var. mysorensis* |
| --- | --- | --- | --- |
| Type form | 0 |  |  |
| Intermediate | -0.00691 | 0 |  |
| *var. mysorensis* | -0.01388 | -0.01392 | 0 |

*p* values non-significant
