# Supplementary material for: Evaluation of intron-1 of odorant-binding protein-1 of Anopheles stephensi as a marker for the identification of biological forms or putative sibling species
Source: PLoS One. 2022 Jul 21;17(7):e0270760. doi: 10.1371/journal.pone.0270760 (PMC9302840; doi:10.1371/journal.pone.0270760)
Supplement: S3 Table — Values above diagonal are pairwise differences between populations (PiXY), diagonal elements are pairwise differences within population (PiX) and below diagonal are corrected pairwise difference (PiXY-(PiX+PiY)/2). (DOCX) [file pone.0270760.s003.docx]

**S3** **Table.** Average pairwise differences between and within biological forms. Values above diagonal are pairwise differences between populations (PiXY), diagonal elements are pairwise differences within population (PiX) and below diagonal are corrected pairwise difference (PiXY-(PiX+PiY)/2)

|  | Type form | Intermediate | *var. mysorensis* |
| --- | --- | --- | --- |
| Type form | 13.58983 | 13.77576 | 14.19657 |
| Intermediate | -0.09492 | 14.15152 | 14.47504 |
| *var. mysorensis* | -0.20441 | -0.20677 | 15.21212 |

*p* values non-significant
