# Supplementary material for: Evaluation of intron-1 of odorant-binding protein-1 of Anopheles stephensi as a marker for the identification of biological forms or putative sibling species
Source: PLoS One. 2022 Jul 21;17(7):e0270760. doi: 10.1371/journal.pone.0270760 (PMC9302840; doi:10.1371/journal.pone.0270760)
Supplement: S1 Fig — The numbers shown above diagonal represent base differences and below diagonal represent genetic distances. (PDF) [file pone.0270760.s004.pdf]

	H1	H10	H2	H3	H12	H4	H6	H11	H15	H8	H14	H9	H13
H1		1	1	2	2	3	9	9	10	16	15	16	14
H10	0.01		0	1	1	4	10	10	11	17	16	17	14
H2	0.01	0		1	1	4	9	9	10	17	16	15	12
H3	0.02	0.01	0.01		2	5	11	11	12	18	17	18	14
H12	0.02	0.01	0.01	0.02		5	10	10	11	16	15	14	11
H4	0.03	0.03	0.04	0.04	0.04		11	11	12	18	17	18	16
H6	0.08	0.09	0.08	0.10	0.09	0.10		1	1	19	18	19	16
H11	0.08	0.09	0.08	0.10	0.09	0.10	0.01		2	20	19	19	16
H15	0.09	0.10	0.09	0.11	0.10	0.11	0.01	0.02		20	19	20	17
H8	0.15	0.16	0.16	0.17	0.15	0.17	0.18	0.19	0.19		1	13	9
H14	0.14	0.15	0.15	0.16	0.14	0.16	0.17	0.18	0.18	0.01		12	8
H9	0.16	0.17	0.15	0.18	0.14	0.18	0.19	0.19	0.20	0.12	0.11		2
H13	0.17	0.17	0.16	0.17	0.14	0.20	0.21	0.21	0.22	0.11	0.09	0.02	
