# Supplementary figures and images for: Evaluation of intron-1 of odorant-binding protein-1 of Anopheles stephensi as a marker for the identification of biological forms or putative sibling species
Source: PLoS One. 2022 Jul 21;17(7):e0270760. doi: 10.1371/journal.pone.0270760 (PMC9302840; doi:10.1371/journal.pone.0270760)

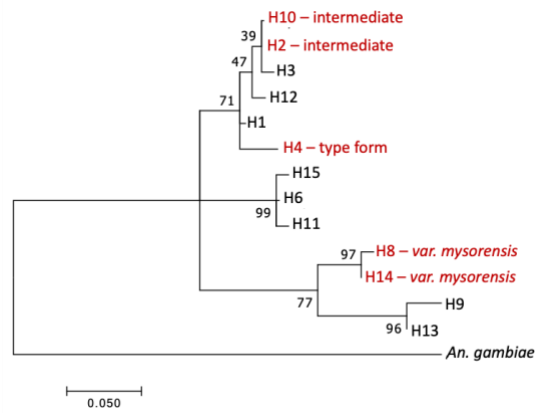

A: Maximum-Likelihood tree

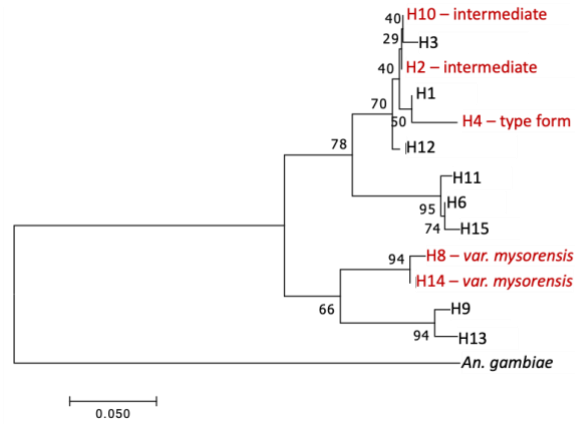

B: Neighbour-Joining tree

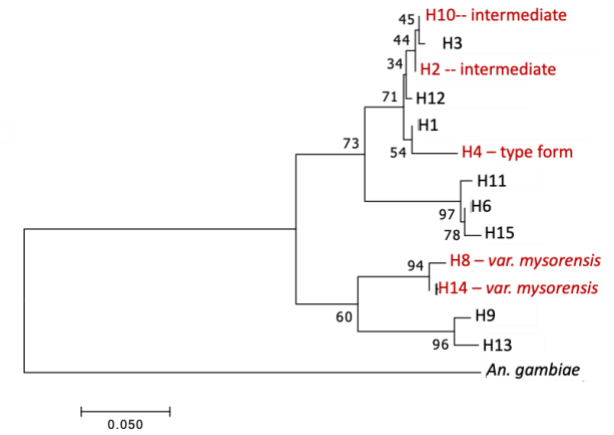

C: Minimum-Evolution tree

Supplement: S2 Fig — The value on each node represent bootstrap value. The haplotypes labelled with red colour font are markers assigned for the identification of haplotypes by earlier workers. The corresponding intron of An. gambiae was taken as outgroup. (PDF) [file pone.0270760.s005.pdf]
