# Supplementary material for: Evaluation of intron-1 of odorant-binding protein-1 of Anopheles stephensi as a marker for the identification of biological forms or putative sibling species
Source: PLoS One. 2022 Jul 21;17(7):e0270760. doi: 10.1371/journal.pone.0270760 (PMC9302840; doi:10.1371/journal.pone.0270760)
Supplement: S1 File — (PDF) [file pone.0270760.s006.pdf]

S1 File. Appendix

Expected heterozygosity (H_E) for n alleles
$H_E = 1-\sum_{i=1}^{n}(pi)\sum_{i=1}^{n}(pi)^2$
(Where *pi* = frequency of ith allele of n alleles)

Maximum expected heterozygosity (H_{E_Max}) will be when alleles are equally frequent (i.e., *pi* = 1/n) in a population
Then,
$H_{E\_Max} = 1-(n(1/n)^2)$
$= 1-(1/n)$
